# Distinct repertoires of microRNAs present in mouse astrocytes compared to astrocyte-secreted exosomes

**DOI:** 10.1371/journal.pone.0171418

**Published:** 2017-02-02

**Authors:** Ana Jovičić, Aaron D. Gitler

**Affiliations:** Department of Genetics, Stanford University School of Medicine, Stanford, CA, United States of America; Martin Luther University, GERMANY

## Abstract

**Background:**

Astrocytes are the most abundant cell type in the central nervous system (CNS) and secrete various factors that regulate neuron development, function and connectivity. microRNAs (miRNAs) are small regulatory RNAs involved in posttranslational gene regulation. Recent findings showed that miRNAs are exchanged between cells via nanovesicles called exosomes. In this study, we sought to define which miRNAs are contained within exosomes secreted by astrocytes. We also explored whether astroglial miRNA secretion via exosomes is perturbed in a mouse model of amyotrophic lateral sclerosis (ALS), a neurodegenerative disease where astrocytes play a crucial role in driving disease progression.

**Methodology/Principal findings:**

By isolating and profiling the expression of miRNAs from primary mouse astrocytes and from the exosomes that astrocytes secrete, we compared miRNA expression in the cells and secreted vesicles. We established that miRNA expression profiles of astrocytes and their exosomes are vastly different. In addition, we determined that exosomal miRNA expression in astrocytes is not significantly perturbed in a mouse model of ALS.

**Conclusions:**

Astrocytes secrete numerous miRNAs via exosomes and miRNA species contained in exosomes are considerably different from miRNAs detectable in astrocytes, suggesting the existence of a mechanism to select certain miRNAs for inclusion or exclusion from exosomes. The exosomal miRNA profiling dataset we have generated will provide a resource to aid in the investigation of this selection mechanism. Finally, the miRNA expression profile in astrocyte-secreted exosomes is not perturbed by expression of mutant SOD1-G93A.

## Introduction

Exosomes are small (50–100 nm) membrane-bound vesicles released by most cells into the extracellular environment and are involved in cell-to-cell communication [1]. Exosomes are formed through inward budding of endosomal membranes, which results in formation of intracellular multivesicular bodies (MVBs). When MVBs fuse with the plasma membrane, exosomes are released into the extracellular environment. Exosomes contain lipids, proteins, RNAs (including microRNAs) and DNA [2; 3]. microRNAs (miRNAs) are 21 to 24 nucleotide long single-stranded RNAs involved in post-transcriptional gene silencing [4, 5]. They bind to messenger RNAs (mRNAs) using sequence complementarity and lead to their degradation or translational repression [6]. Because miRNAs pair imperfectly with their mRNA targets they have a potential to regulate hundreds of different transcripts [7]. miRNAs can be transferred from one cell to the other via exosomes, which enables them to potentially exert a broad influence at the tissue and organism level [3]. In the recipient cells miRNAs readily bind local mRNAs and actively regulate translation [8, 9]. Therefore, exosome-mediated regulation of translation in the recipient cells represents a powerful and versatile tool for establishing different translational programs. An important step towards understanding potential roles of exosome-mediated translational regulation is deciphering exactly which miRNAs are present in exosomes originating from a particular cell type.

Virtually all cells secrete exosomes, including neurons [10], astrocytes [11] and myelinating cells [12, 13]. Astrocytes are the most abundant cell type in the central nervous system and perform multiple and diverse roles. They interact with neurons and vasculature to provide metabolic support to neurons [14], remove and recycle neurotransmitters from the synaptic cleft and secrete numerous factors that control synapse formation and function [15, 16]. Recently it has been demonstrated that astrocytes secrete exosomes containing miR-19a, which regulates PTEN expression in brain tumor cell, thus influencing the outgrowth of brain metastases [11]. In this study we sought to define the miRNA expression profile of astrocyte-derived exosomes. In addition, do the miRNAs present in exosomes directly correlate with their abundance in astrocytes or are certain astrocyte-derived miRNAs preferentially packaged into exosomes whereas others are not? Finally, because of the prominent cell non-autonomous disease mechanisms in ALS [17], we compared the miRNAs expressed in wild type or mutant SOD1-expressing mouse astroglial exosomes. We present our results as a comprehensive resource for future studies aimed at elucidating specific roles of miRNAs secreted by astroglial exosomes.

## Results

### miRNA expression profile of primary mouse astrocytes

**Fig 1:**
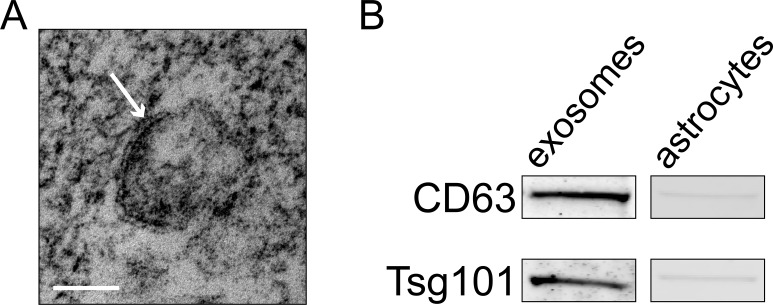
Characterization of extracellular vesicles isolated from astrocyte-conditioned media. A) Transmission electron microscopy revealed presence of exosomes, characterized as double membrane-bound vesicles (indicated by arrow), between 50 and 100 nm in size. Scale bar = 50 nm. B) Western blot analysis confirmed expression of CD63 and Tsg101 in astroglial exosomes, while astrocytes showed very low levels of expression of these proteins.

**Fig 2:**
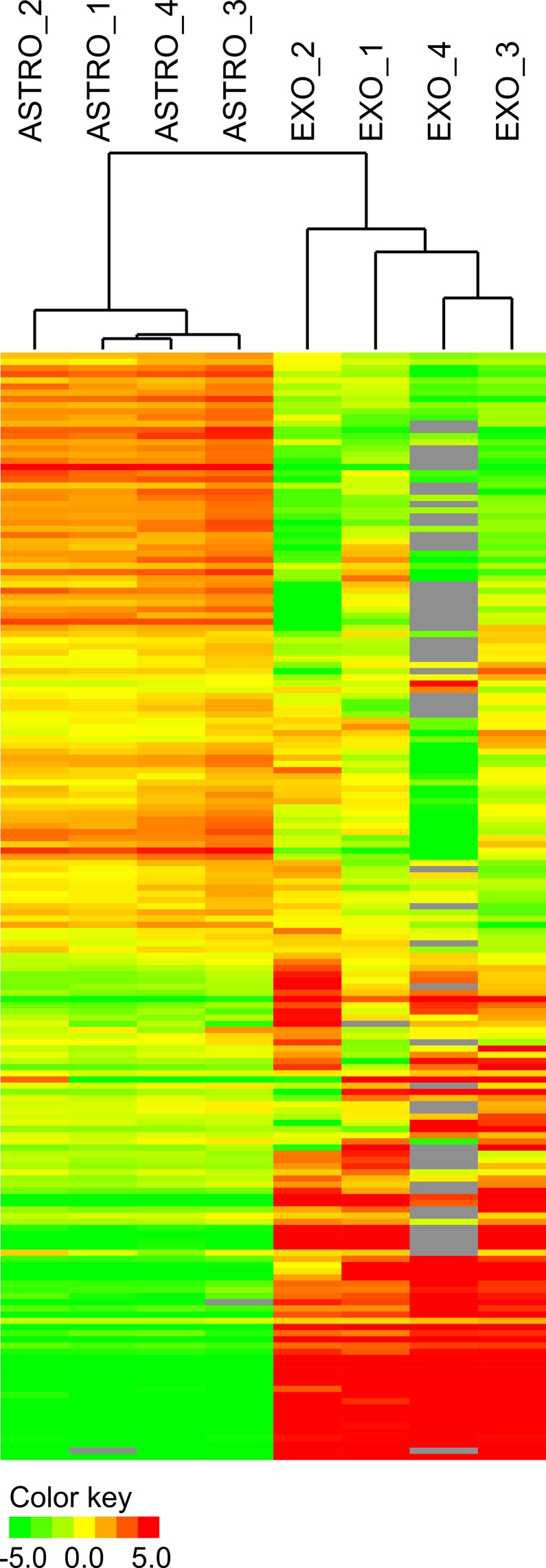
Astrocytes and astrocyte-secreted exosomes differ vastly in their miRNA expression. ASTRO_1, 2, 3, 4 –astrocyte samples; EXO_1, 2, 3, 4 –exosome samples.

The central question we sought to answer was which miRNAs are contained within astrocyte-secreted exosomes. In order to purify astroglial exosomes, we prepared primary mouse astrocytes as previously described [18]. After 10 days *in vitro* exosomes were purified from the astrocyte-conditioned media by a series of centrifugation and filtration steps [19]. Identity of the isolated vesicles was confirmed using transmission electron microscopy, which revealed 50–100 nm double membrane-bound vesicles (Fig 1A). In addition, the isolated vesicles expressed established exosomal markers CD63 and Tsg101 (Fig 1B). Total RNA was isolated from both the exosomes and primary astrocytes and miRNA expression was profiled using rodent TaqMan miRNA arrays. We quantified the expression of 752 mature rodent miRNAs in 4 independently prepared primary astrocyte and exosome RNA samples. Expression profiling showed that astroglial exosomes have a substantially different miRNA content compared to astrocytes (Fig 2). We provide the full dataset of miRNA expression in S1 and S2 Tables. Hierarchical clustering clearly separated the 4 exosomal samples from the 4 astroglial samples. 217 miRNAs were exclusively detected in astrocytes, while 61 miRNAs were more than 2-fold enriched in astrocytes compared to exosomes (Table 1). Expression of miRNAs in exosomes was much more restrictive–only 12 miRNAs were exclusively present in exosomes, and 42 miRNAs had higher than 2-fold enrichment in exosomes compared to astrocytes (Table 2). To validate these data we performed qPCR with pre-amplification cycle for two exosome-specific miRNAs, miR-10b and miR-208. While we were unable to detect them in astrocytes, we readily detected them in exosomes. On the other hand, miR-188-5p which is astrocyte-specific was detected in astrocytes, but not in exosomes (Table 3). These findings suggest that specific miRNAs are selectively recruited to exosomes, as miRNAs in exosomes were vastly different from miRNAs in astrocytes. Deciphering the cellular mechanisms regulating this selective recruitment of specific astroglial miRNAs to exosomes will be the subject of future studies.

**Table 1:** miRNAs enriched in astrocytes compared to exosomes.

miRNAs	Fold enrichment (astrocytes/exosomes)
let-7a, let-7a*, let-7c-1*, let-7e-3p, let-7f, let-7g*, let-7i-3p, miR-1, miR-7a-1-3p, miR-7b, miR-15a-3p, miR-16-1-3p, miR-18a-3p, miR-20a-3p, miR-21-3p, miR-22, miR-24-2-5p, miR-26b-3p, miR-27a-5p, miR-27b-5p, miR-28a-3p, miR-29a-5p, miR-29c-5p, miR-30b-3p, miR-30d-3p, miR-31-3p, miR-32, miR-33-3p, miR-99a, miR-99b-3p, miR-101a, miR-101b, miR-106b-3p, miR-107, miR-125a-3p, miR-125b-2-3p, miR-125b-3p, miR-129-5p, miR-130b-5p, miR-132, miR-134, miR-135b, miR-136, miR-137, miR-138, miR-140-3p, miR-141, miR-143, miR-146b, miR-148a, miR-148b, miR-151-3p, miR-151-5p, miR-153, miR-154, miR-154-3p, miR-155, miR-181c, miR-182, miR-187, miR-188-5p, miR-190b, miR-191-3p, miR-193a-5p, miR-196c, miR-197, miR-199b, miR-200b, miR-200c, miR-200c, miR-202-3p, miR-202-5p, miR-205, miR-207, miR-210, miR-213, miR-214, miR-215, miR-218-1-3p, miR-219, miR-224, miR-292-3p, miR-296-3p, miR-298, miR-299-5p, miR-322, miR-323-3p, miR-324-3p, miR-325, miR-329, miR-335-3p, miR-337-3p, miR-338, miR-338-3p, miR-338-5p, miR-339-3p, miR-339-5p, miR-340, miR-342-5p, miR-344, miR-345-3p, miR-34b, miR-34b-5p, miR-34c-3p, miR-350, miR-351, miR-352, miR-362-5p, miR-365, miR-369-5p, miR-370, miR-376a, miR-376a-5p, miR-376b, miR-376b-5p, miR-380-3p, miR-380-5p, miR-383, miR-384-3p, miR-409-3p, miR-409-5p, miR-411-3p, miR-423-3p, miR-425, miR-429, miR-431, miR-433, miR-434-5p, miR-449b, miR-450a, miR-450a-5p, miR-450b-3p, miR-455, miR-463, miR-465a-5p, miR-465c-3p, miR-466g, miR-466k, miR-467a, miR-467b, miR-467c, miR-467d, miR-467e, miR-485-3p, miR-487b, miR-488, miR-491, miR-497, miR-500, miR-503, miR-503-3p, miR-532-5p, miR-540-3p, miR-541, miR-542-3p, miR-542-5p, miR-543, miR-547, miR-582-3p, miR-582-5p, miR-592, miR-598, miR-632, miR-667, miR-668, miR-669a, miR-669c, miR-669h-5p, miR-669m, miR-669n, miR-669o, miR-670, miR-672, miR-673, miR-673-3p, miR-674, miR-674-3p, miR-676, miR-676-5p, miR-677, miR-690, miR-700, miR-701, miR-706, miR-708, miR-758, miR-872, miR-873, miR-877-3p, miR-878-3p, miR-879-3p, miR-881-5p, miR-1306, miR-1839-5p, miR-1894-3p, miR-1896, miR-1897-3p, miR-1897-5p, miR-1193, miR-1198, miR-1903, miR-1904, miR-1905, miR-1932, miR-1935, miR-1941-5p, miR-1943, miR-1944, miR-1949, miR-1953, miR-1954, miR-1968, miR-1969, miR-1971, miR-1981, miR-1982.2, miR-2183, snoRNA135, snoRNA135, snoRNA202	N/A (detected in astrocytes, not detected in exosomes)
let-7b, let-7c, let-7d, let-7e, let-7g, let-7i, miR-9, miR-16, miR-20b, miR-23b, miR-26a, miR-29a, miR-30c, miR-30e, miR-34b-3p, miR-34c, miR-99a, miR-99b, miR-100, miR-103, miR-125a-5p, miR-125b-5p, miR-129-3p, miR-135a, miR-139-5p, miR-140, miR-143, miR-145, miR-181a, miR-193b, miR-199a-3p, miR-204, miR-221, miR-296-5p, miR-301a, miR-301b, miR-320, miR-324-5p, miR-331-3p, miR-335-5p, miR-340-3p, miR-340-5p, miR-342-3p, miR-350, miR-361, miR-375, miR-379, miR-382, miR-384-5p, miR-411, miR-434-3p, miR-449a, miR-487b, miR-494, miR-495, miR-543, miR-574-3p, miR-671-3p, miR-744, miR-872-3p, miR-1839-3p, snoRNA202	>2

**Table 2:** miRNAs enriched in exosomes compared to astrocytes.

miRNAs	Fold enrichment (exosomes/astrocytes)
miR-10b, miR-96, miR-196a*, miR-208, miR-505, miR-547, miR-590-5p, miR-697, miR-685, miR-715, miR-1195, miR-1959	N/A (detected in exosomes, not detected in astrocytes)
miR-10a, miR-20a, miR-25, miR-92a, miR-106b, miR-148b-5p, miR-192, miR-685, miR-715, miR-2134	> 2
miR-126-5p, miR-345, miR-367, miR-652, miR-1937b, miR-1937c	>100
miR-150, miR-184, miR-186-3p, miR-189, miR-190b, miR-194, miR-206, miR-302a, miR-302b, miR-374-5p, miR-378, miR-381, miR-410, miR-421, miR-451, miR-544, miR-546, miR-551b, miR-671-5p, miR-696, miR-704, miR-712, miR-721, miR-805, miR-1274a, miR-2182	>1000

**Table 3:** qPCR confirmation of exosome-specific expression on miR-10b and miR-208.

miRNA	Cycle threshold in exosomes	Cycle threshold in astrocytes
miR-10b	21.92	undetermined
miR-208	25.43	undetermined
miR-188-5p	undetermined	15.99

In addition to miRNAs, we serendipitously identified some tRNA fragments within astroglial exosomes. miR-1274a, miR-1937b, miR-1937c, miR-2134 and miR-2182 were included in the commercial TaqMan rodent miRNA arrays that we used but turn out to actually represent tRNA fragments instead of mature miRNAs [20]. These tRNA fragments are not random by-products of tRNA degradation or biogenesis and have biological regulatory roles [21]. Interestingly, we found all of these regulatory tRNA fragments to be highly enriched in exosomes (Table 1). This raises a question of the full repertoire of non-coding RNAs present in exosomes and underlies the regulatory potential of exosomes.

### The profile of exosomal miRNAs is not changed in mouse SOD1-G93A ALS astrocytes

ALS is a progressive neurodegenerative disease with a strong non-cell autonomous component contributing to motor neuron death [17]. Selectively deleting mutant SOD1 transgene expression in astrocytes, sharply slows the disease progression in the SOD1 mouse model of ALS [22]. Moreover, astrocytes seem to secrete toxic factor(s) that are detrimental to motor neuron health [23–26]. Given the implication of astrocyte-secreted factors in neurodegeneration, we considered the possibility that abnormal exosome-mediated secretion of miRNAs from astrocytes might be a component of the non-cell autonomous toxicity. Therefore, we sought to define the exosome miRNA expression profile in SOD1-G93A mouse astrocytes.

**Fig 3:**
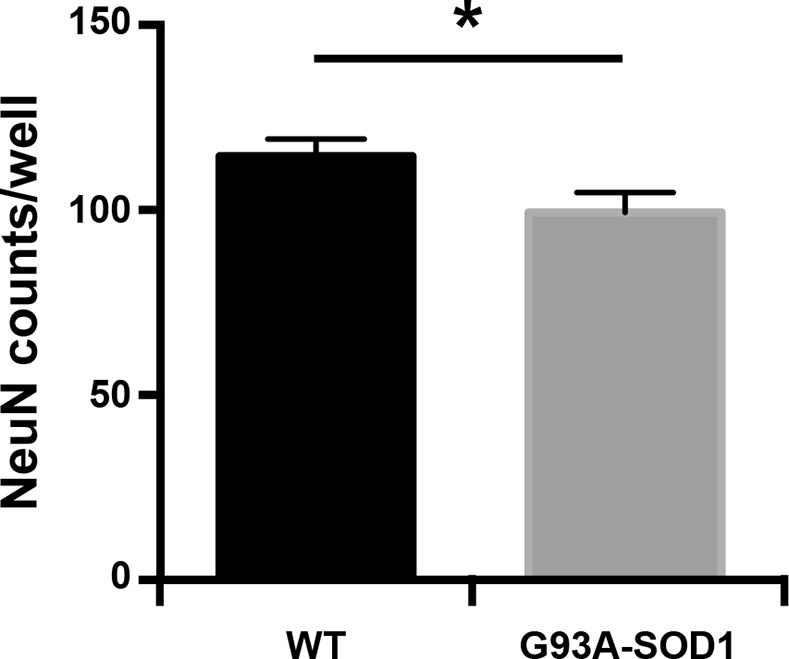
Conditioned media from SOD1-G93A astrocytes is toxic to motor neurons. Astrocyte-conditioned media was added to primary motor neurons and their viability was assessed after 7 days using anti-NeuN antibody (Student’s t-test, p<0.01).

We collected the media from primary astrocytes prepared from the SOD1-G93A mouse model of ALS and from wild type littermates. Consistent with previous studies [23–26], we validated that the conditioned media from these cultures is toxic to motor neurons. Treating primary spinal cord neurons with SOD1-G93A astrocyte-conditioned media significantly decreased neuronal viability after 3 days compared to the media from wild-type astrocytes (Fig 3). However, expression profiling of exosomal miRNAs in SOD1-G93A and wild type astrocytes did not show any significant differences. Therefore, the observed toxicity of SOD1 astrocyte media is likely to not be mediated by differential expression of profiled exosomal miRNAs. However, as we have not performed expression profiling of all mouse miRNAs, but only the 752 mature miRNAs present on commercial TaqMan arrays, we cannot exclude a possibility that some of the miRNAs not present on the arrays might play a role in astrocyte toxicity. More importantly, whether exosomes, either through their RNA, DNA, protein or lipid content, contribute to SOD1-G93A astrocyte-mediated toxicity remains to be further examined. Interestingly, increased exocytosis has been observed in SOD1-G93A astrocytes and it contributes to the astrocyte-mediated toxicity [27]. Whether this results in increased abundance of secreted exosomes should be determined in future studies.

## Discussion

In the present study we have confirmed that primary astrocytes secrete miRNA-containing exosomes [11]. The profile of miRNAs in exosomes secreted from astrocytes is remarkably different from the profile of miRNAs in astrocytes. miRNAs enriched in astrocyte exosomes have not been previously extensively studied with respect to their roles in the brain. Nevertheless, there is some evidence that perturbation of their expression can have wide-ranging consequences to central nervous system development or function. For example, miR-302, which we have found to be highly enriched in exosomes, has been shown to regulate neural progenitor proliferation, differentiation timing, and survival during neurulation, and knockout or miR-302 results in early embryonic lethality and open neural tube defects [28]. On the other hand, several of the exosomal miRNAs that we have identified have been implicated in various disorders: miR-297a has been found to be upregulated in the post-ischemic cortex at 24h [29], miR-1928 is a stress-responsive miRNA in amygdala and may serve as a biomarker for posttraumatic stress disorder [30], while miR-432 might represent a biomarker for schizophrenia [31, 32].

Given that numerous miRNAs are detected in the exosomal fraction, the question remains whether they are all detected in each exosome secreted by astrocytes or are there different subpools of exosomes each containing its own miRNA repertoire? Similarly, an outstanding question is whether all astroglial exosomes are targeted to the same recipient cell type or different ones, and which cell types would these be? It has already been shown that oligodendrocytes can transfer exosomes to neurons [12]. Could astrocytes also participate in the local regulation of neuronal translation by delivering pertinent miRNAs? Astrocytes are in close proximity to neuronal postsynaptic compartments and have already been shown to perform crucial roles required for synaptic function [33].

We present the current dataset as a resource for future studies. We have extensively characterized the miRNA content of mouse astrocyte-secreted exosomes and showed that exosomes contain a specific repertoire of miRNAs, which is surprisingly different from the repertoire of cellular miRNAs present in astrocytes. Further investigations into which cells are these exosomes targeted to and which mRNAs they regulate will undoubtedly bring us closer to better understanding mechanisms of exosome-mediated intercellular communication in the central nervous system.

## Materials and methods

### Primary mouse astroglial culture

Dissociated glial cultures were prepared from cortexes of postnatal day 1 (P1) mice (of both sexes), using Worthington Papain Dissociation System following manufacturers protocol. To obtain glial cells, mixed cortical cultures were grown in Dulbecco’s Modified Eagle Medium (DMEM, Gibco) supplemented with 10% exosome-depleted fetal bovine serum (Gibco), 1× penicillin-streptomycin (Gibco). After 10–14 d in vitro, microglial cells and oligodendrocyte precursors were eliminated by shaking on a rotary shaker at 300 rpm for 3 h, after which the medium was replaced. Astrocytes were cultured for additional 10 days, after which media was collected for exosome purification. At the same time point, the remaining astrocytes on the dish were washed with PBS 3 times, lysed and their RNA was extracted.

### Primary mouse motor neuron culture

All mouse experiments were performed in compliance with Stanford Administrative Panel on Laboratory Animal Care guidelines and regulations. Stanford Administrative Panel on Laboratory Animal Care specifically approved this study. Dissociated primary motor neuron cultures were prepared from spinal cords of embryonic day 12.5 mice, using Worthington Papain Dissociation System following manufacturers protocol. Neurons were cultured in Neurobasal Medium (Gibco), supplemented with 1x B27 (GIbco), 2% horse serum, 0.5 mM L-glutamine, 1ng/ml BDNF, 100 pg/ml GDNF and 10 ng/ml CTNF.

### Exosome purification

Exosomes were prepared from the astrocyte-conditioned media by differential centrifugation as previously described [3]. Briefly, media was collected, centrifuged at 2000*g* for 10 min to eliminate dead cells and at 16,500*g* for 20 min to eliminate cell debris, followed by filtration through a 0.22 μm filter. Exosomes were pelleted by ultracentrifugation at 120,000*g* for 90 min, pellet was washed once with PBS and ultracentrifuged again.

In order to eliminate serum-derived exosome contamination in the media, before use fetal bovine serum was ultracentrifuged at 120,000*g* for 90 min.

Astrocytes were cultured in 75 cm^2^ flasks and for a single exosome isolation, conditioned media from two flasks would be pooled together.

### RNA isolation, quantitative PCR and qPCR arrays

Total RNA was isolated using mirVana microRNA isolation kit (Ambion). miRNA assays were conducted using TaqMan miRNA assay kits or TaqMan rodent microRNA A + B cards set v3.0 (Applied Biosystems). Hierarchical clustering was performed using Cluster 3.0 [34].

### Western blotting

Proteins were isolated form exosomal fraction by adding SDS sample buffer and heating samples to 70°C for 10 min. Total of 7.5 μg of proteins was loaded in a single well and separated by SDS-PAGE and transferred onto a nitrocellulose membrane. After membranes were blocked for one hour at room temperature in Odyssey Blocking Buffer (LI-COR Biosciences), followed by incubation in primary antibodies diluted in Blocking Buffer with 0.1% Tween-20 overnight at 4°C. Membranes were rinsed with PBS followed by incubation with secondary antibody diluted in Blocking Buffer for one hour at RT. After final rinses, blots were scanned with Odyssey Infrared Imager (LI-COR Biosciences). Primary and secondary antibodies comprised: anti-CD63 (Santa Cruz Biotechnology, sc-15363; 1:1000), anti-Tsg101 (Genetex, gtx70255; 1:1000), goat anti-mouse Alexa Fluor 680 (Thermo Fisher Scientific, A-21058; 1:15,000), goat anti-rabbit Alexa Fluor 790 (Thermo Fisher Scientific, A-11369; 1:15,000).

### Electron microscope analysis of whole-mount exosomes

Exosome-containing pellet was resuspended in 50 μl of 2% paraformaldehyde. 5 μl of the resuspended pellets was deposited on Formvar-carbon coated electron microscopy grids. Membranes were covered and let to adsorb for 20 min in a dry environment. After washing the samples on drops of PBS, samples were incubated on drops of 1% gluteraldehyde for 5 min, and then washed eight times on drops of distilled water. Afterwards, samples were contrasted first in a solution of uranyl-oxalate solution, pH 7, for 5 min, and then contrasted and embedded in a mixture of 4% uranyl acetate and 2% methyl cellulose in a ratio of 100μl/900μl respectively, for 10 min on ice. Stain was blotted dry from the grids with filter paper and samples were allowed to dry. Samples were then examined under electron microscope at 80 kV.

## Supporting information

S1 Table miRNA expression levels in primary astrocytes. miRNA expression was assayed in 4 separately prepared primary mouse astrocytes using TaqMan microRNA cards A and B and original Ct values are reported in the table.
(XLSX)

S2 Table miRNA expression levels in astroglial exosomes. miRNA expression was assayed in 4 separately prepared exosome-derived RNA samples using TaqMan microRNA cards A and B and original Ct values are reported in the table.
(XLSX)
